# Differential Expression ESTs Associated with Fluorosis in Rats Liver

**DOI:** 10.1155/2012/208390

**Published:** 2012-02-13

**Authors:** Y. Q. He, Y. Pan, L. J. Ying, R. Zhao

**Affiliations:** ^1^The Laboratory Animal Research Center, Jiangsu University, Zhenjiang 212013, China; ^2^College of Food Science and Biological Engineering, Jiangsu University, Zhenjiang 212013, China; ^3^School of Clinical Medicine, Jiangsu University, Zhenjiang 212013, China

## Abstract

The fluoride has volcanic activity and abundantly exists in environment combining with other elements as fluoride compounds. Recent researches indicated that the molecular mechanisms of intracellular fluoride toxicity were very complex. However, the molecular mechanisms underlying the effects on gene expression of chronic fluoride-induced damage is unknown, especially the detailed regulatory process of mitochondria. In the present study, we screened the differential expression ESTs associated with fluorosis by DDRT-PCR in rat liver. We gained 8 genes, 3 new ESTs, and 1 unknown function sequence and firstly demonstrated that microsomal glutathione S-transferase 1 (MGST1), ATP synthase H^+^ transporting mitochondrial F_0_ complex subunit C1, selenoprotein S, mitochondrial IF1 protein, and mitochondrial succinyl-CoA synthetase alpha subunit were participated in mitochondria metabolism, functional and structural damage process caused by chronic fluorosis. This information will be very helpful for understanding the molecular mechanisms of fluorosis.

## 1. Introduction

The fluoride has volcanic activity and abundantly exists in environment combining with other elements as fluoride compounds. With the development of the world economy, more and more organofluorine compounds are increasingly used. These compounds have a wide range of functions and can serve as agrochemicals, pharmaceuticals, refrigerants, pesticides, surfactants, fire-extinguishing agents, fibers, membranes, ozone depletors, and insulating materials [1, 2]. At the same time, in last decade, the fluoride effects have resurfaced due to the awareness that this element interacts with cellular systems even at low doses. Excessive fluoride intake over a long period of time may result in a serious public health problem called fluorosis, which is characterized by dental mottling and skeletal manifestations such as crippling deformities, osteoporosis, and osteosclerosis [3].

In recent years, metabolic, functional, and structural damage caused by chronic fluorosis have been reported in many tissues. Fluoride can induce oxidative stress and modulate intracellular redox homeostasis, lipid peroxidation, and protein carbonyl content, as well as alter gene expression and cause apoptosis. Genes modulated by fluoride include those related to the stress response metabolic enzymes, the cell-cell communications, and signal transduction [1]. In most cases, fluoride acts as an enzyme inhibitor, but fluoride ions can occasionally stimulate enzyme activity. Research data strongly suggest that fluoride inhibits protein secretion and/or synthesis and that it influences distinct signaling pathways involved in proliferation and apoptosis including the mitogen-activated protein kinase (MAPK), p53, activator protein-1 (AP-1), and nuclear factor kappa B (NF-*κ*B) pathways [4–6]. However, information about the molecular mechanism of fluoride-induced tissue damage is almost unknown. Some studies point out that the mitochondria are the key intracellular targets for different stressors including fluoride [7]. Fluoride alters the activity of many mitochondria-rich cells such as those of the human kidney and the rat liver [8, 9], but the molecular mechanisms of chronic fluoride-induced mitochondrial damage are scarce. As an important detoxification organ, liver plays a pivotal role on resolving fluorosis effects. In the present study, in order to get a deeper understanding of the molecular mechanisms underlying the effects of fluoride on mitochondrial gene expression and metabolism, we screened differential display genes or expressed sequence tags (ESTs) involved in Sprague-Dawley rats with fluorosis.

## 2. Materials and Methods

### 2.1. Animals and Fluoride Exposures

The protocol for the study was reviewed and approved by the Institutional Animal Use and Care Committees of Jiangsu University. The male Sprague-Dawley rats (*n* = 30) were received three week after weaning, an age at which all regions of the central nervous system are rapidly developing. All animals were housed in stainless-steel cages suspended in stainless-steel racks with the humidity ranged from 30 to 55%, and the room temperature remained between 22 and 25°C, fed with a standard pellet diet, and given distilled water ad libitum. The animals were allowed to acclimatize to the laboratory conditions for four days before experiments began.

The rats were weighed and randomly divided into 2 groups (15/group). The first group animals were injected i.p. with aqueous NaF (20 mg/kg/body weight/day) selected on the basis of the LD50 value of fluoride, which is 51.6 mg/kg body weight/day in mice and maintained for 30 days. The second group served as control and was injected mammalian physiological saline. The NaF solution (30 mL) was prepared fresh each day in double distilled water. At the end of the 30-day treatment, the animals were killed by decapitation, the brains were rapidly dissected, and the hippocampus removed. The tissue was immediately frozen in liquid nitrogen and stored at −80°C until use. The experiments were performed in accordance with the regional legal regulations. The body weight of each animal was weighed before treatment, and weighed every 10 days. The F-levels in serum were determined [10] by using a CSB-F-I fluoride ion electrode (Changsa Analysis Instrumentation, China) to confirm chronic fluorosis.

### 2.2. Total RNA Extraction and RNA Pools

 Total RNA of liver samples was extracted using Trizol kit (Invitrogen, Carlsbad, USA) according to the manufacturer's instructions. To remove any contaminated genomic DNA from the RNA samples, 25 *μ*L of total RNA was treated with an equal volume of RNase-free DNase-I mixture (Promega, Madison, WI, USA), and the whole mixture was incubated at 37°C for 30 min. Subsequently, the mixture was subjected to phenol-chloroform extraction followed by ethanol precipitation. The concentration of purified RNA was determined by absorbance at 260 nm. An RNA pool containing equal amount RNA of three individuals from the same breed was established. The RNA samples were stored at −70°C.

### 2.3. Primers

The cDNA primers of three anchored primers of H-T_11_A, H-T_11_G and H-T_11_C and eight arbitrary primers of P1–P8 were designed according to the third generation primers of GenHunter Company. The 3′ end anchored primers were

H-T_11_G: 5′-AAGCTTTTTTTTTTTG-3′;

H-T_11_C: 5′-AAGCTTTTTTTTTTTC-3;

H-T_11_A: 5′-AAGCTTTTTTTTTTTA-3.

The 5′ end arbitrary oligonucleotide primers were:

P1: 5′-TGCCGAAGCTTTGGTGTC-3′;

P2: 5′-TGCCGAAGCTTTGGTACC-3′;

P3: 5′-TGCCGAAGCTTTGGTAGC-3′;

P4: 5′-TGCCGAAGCTTTGGTATG-3′;

P5: 5′-TGCCGAAGCTTTGGTCAC-3′;

P6: 5′-TGCCGAAGCTTTGGTCAG-3′;

P7: 5′-TGCCGAAGCTTTGGTCTG-3′;

P8: 5′-TGCCGAAGCTTTGGTCTC-3′.

### 2.4. Reverse Transcription

A 1 *μ*L of 3′ anchored primer (1 *μ*mol/L) was combined with 4 *μ*L (1 *μ*g/*μ*L) RNase-free DNase I-treated RNA samples and 1 *μ*L of 10 mmol/L dNTP mix, the mixture was adjusted to a final volume of 11 *μ*L by adding DEPC-treated water. Then it was incubated for 5 min at 65°C followed by cooling on ice for 2 min. First-strand synthesis was initiated by mixing the mixture with 1 *μ*L (50 units/*μ*L) M-MLV reverse transcriptase (Invitrogen) in a final volume of 8 *μ*L mix containing 2 *μ*L of 10 × RT buffer (200 mmol/L Tris-HCl (pH 8.4), 500 mmol/L KCl), 4 *μ*L of 25 mmol/L MgCl_2_, and 2 *μ*L of 0.1 mol/L DTT. The reverse transcription reaction was carried out at 42°C for 1 h, then terminated by heating at 70°C in a water bath for 15 min. The reverse transcription product of cDNA was stored at −20°C.

### 2.5. DD-PCR

A 1 *μ*L of the reverse transcription product was added to 24 *μ*L of PCR solution containing 20 *μ*mol/L dNTPs, 1.5 mmol/L MgCl_2_, 1 U of Taq DNA polymerase (Promega), 20 *μ*mol/L H-T_11_N (H-T_11_G/H-T_11_C/H-T_11_A) primer used for RT, and 10 *μ*mol/L of one of eight arbitrary primers. The PCR reaction was carried out on Mastercycler 5333 (Eppendorf AG, Hamburg, Germany). The PCR parameters were as follows: initial denaturation at 94°C for 5 min; followed by one cycle of denaturation at 94°C for 30 s, annealing at 40°C for 2 min, extension at 72°C for 2 min; followed by 35 cycles of denaturation at 94°C for 30 s, annealing at 60°C for 2 min, extension at 72°C for 2 min; with a final extension step at 72°C for 5 min. The amplified fragments of DDRT-PCR were separated by electrophoresis using 8% polyacrylamide gels (acrylamide : bisacrylamide = 39 : 1). Then, the PCR products were visualized by silver staining and photographed and analyzed using an AlphaImager 2200 and 1220 Documentation and Analysis Systems (Alpha Innotech Corporation, San Leandro, CA, USA).

### 2.6. Reverse Northern Dot Blot Analysis

Reverse Northern dot blotting was carried out according to the instruction manual of DIG-HIGH Prime DNA labeling and detection starter kit I (Roche, Penzberg, Germany). In brief, 1 *μ*g of template DNA was denatured by heating in a boiling water bath for 10 min and quickly chilling in an ice/water bath, then DIG-HIGH Prime was mixed and incubated at 37°C for 1 h, and the reaction was stopped by adding 2 *μ*L of 0.2 mol/L EDTA (pH 8.0). Subsequently, the RT products were fixed on the nylon membranes positively charged by UV crosslinking and were then hybridized in DIG Easy Hyb (10 mL/100 cm^2^) containing DIG-labeled DNA probe at appropriate hybridization temperature for 20 h. After hybridization and stringency washes, the nylon membranes were incubated in 100 mL blocking solution for 30 min and in 20 mL antibody solution for 30 min. Then it was detected in freshly color substrate solution in the dark after washing two times in 100 mL washing buffer for 15 min, and the reaction will be completed after 16 h and terminated by 50 mL TE buffer for 5 min.

### 2.7. Purification, Sequencing, and Analysis of Differential Bands

Differential fragments from 100 bp to 1200 bp were excised from the gels, extracted by boiling in 100 *μ*L of distilled water, purified with a DNA purification system (Promega), reamplified using the same primer corresponding to cDNA display. The PCR amplification conditions were as follows: initial denaturation at 94°C for 3 min; followed by 33 cycles of denaturation at 94°C for 2 min, annealing at 55°C for 2 min, extension at 72°C for 2 min; with a final extension at 72°C for 5 min. The 25 *μ*L of the reamplified PCR sample along with 5 *μ*L of gel loading buffer was analyzed on 1.5% agarose/EtBr gel in 1 × TAE (40 mmol/L Tris base, 20 mmol/L acetic acid, 2 mmol/L EDTA pH 8.0). The PCR bands of interest were cut from the gels under UV illumination. The DNA was purified using DNA extraction kit (Qiagen, Hilden, Germany). The purified differential bands were ligated into the pGEM-T Easy vector (Promega) according to manufacturer′s instructions and transformed into *Escherichia coli* DH5*α* competence cells. The positive clones were sequenced using an automatic ABI 3730 sequencer (Perkin Elmer Applied Biosystems, Foster City, CA, USA) by Shanghai Invitrogen Biotechnology Ltd. Co. (Shanghai, China) and analyzed in GenBank by BLAST.

## 3. Results

### 3.1. Extraction and Reverse Transcription of Total RNA

We collected the livers at the same conditions except fluoride treatment for extracting the RNA. The gel profile of RNA showed that two bands of 18S and 28S were clear and could be used for reverse transcription.

### 3.2. DD-PCR

**Figure 1:**
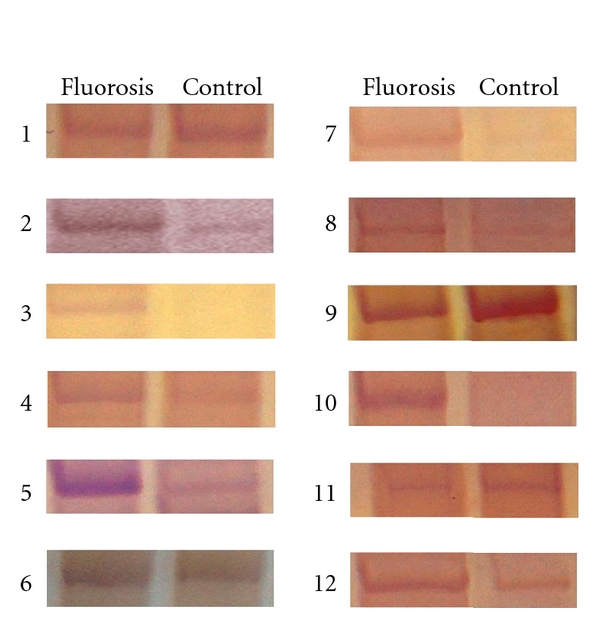
Differential display profiles from different primer combinations of rat liver. 1–12: correspond to the no. of Table 1.

We displayed the different bands of two kinds of cDNA using 24 primer pairs consisting of 3 anchored primers and 8 arbitrary primers. The partial results were shown in Figure 1. At last 1532 bands were detected on statistics, and 36 bands were recovered and sequenced based on their differences (lack, density, and size). The recovered differential bands were reamplified with the same anchor primer and arbitrary primer using the recovered products as templates. However, some of the recovered bands could not be perfectly amplified because of the false positive.

### 3.3. Reverse Northern Dot Blot

**Figure 2:**
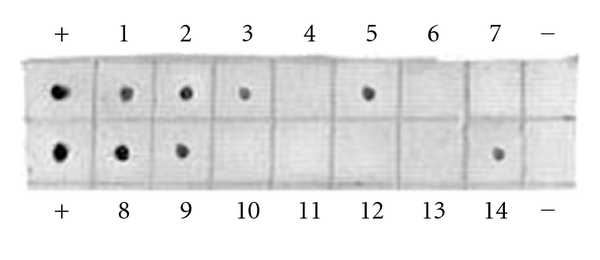
Partial results of reverse northern dot blots for differential display bands. +: positive control; −: false control. 1–14: the results of dot blot of bands, visible dot represents positive result, and no dot represents false result.

In order to eliminate the disruption of false positive bands, reverse northern was carried out to validate differential bands. 36 differential bands of PCR amplifications were blotted on two nylon membranes and hybridized with probes of liver cDNA. The results showed that 13 positive blots were detected (Figure 2).

### 3.4. Sequence Analysis of Differential Bands

All the13 positive bands detected by reverse northern dot blot were sequenced and analyzed in GenBank by BLAST. The alignment standards include that the ESTs length should be more than 100 bp, the *E* value should be less than 0, and the identity should exceed 90%. The results were listed in Table 1. The BLAST results demonstrated that 8 genes including microsomal glutathione S-transferase 1, acyl-CoA synthetase long-chain family member, ATP synthase H^+^ transporting mitochondrial F_0_ complex subunit C1, haptoglobin, selenoprotein S, mitochondrial IF1 protein, acyl-CoA synthetase long-chain family member, mitochondrial succinyl-CoA synthetase alpha subunit, 3 new ESTs, and 1 unknown function sequence were differential expressed.

## 4. Discussion

Several molecular strategies including microarray analysis, in vivo expression technology (IVET), DDRT-PCR, and signature-tagged mutagenesis have been used to identify differential expression genes in all kinds of organs, tissues, and individuals. Although DDRT-PCR was originally known that it had highly false positive bands, recent modifications have allowed this technique to be used again perfectly. In this study, by using a novel and modified DDRT-PCR technique, the primers were the third generation DDRT-PCR primers consisting of both anchored and arbitrary primers, which have Hind enzyme site. The number of primers also reduces to three anchored primers and eight arbitrary primers. This primer combination had proved by computer analysis to possess the ability to include all mRNAs in certain stage of some cell development, which makes it easier for next operation and treatment. As in most described DDRT-PCR protocols, total RNA was used as the starting material. In the present study, to exclude partial degradation, we tested total RNA preparation with DNase I to reduce artifacts caused by possible contamination with genomic DNA. To reduce the rate of mismatches that occur with low stringency annealing during subsequent PCR cycles, we followed the strategy published by Zhao et al. [11], the cycle procedure was only one initial cycle with low stringency annealing temperature (40°C), and followed by 35 high stringency PCR cycles with annealing temperature (60°C). This method significantly improved the reproducibility of band patterns.

According to the results of the sequence BLAST, we gained 8 genes, 3 new ESTs, and 1 unknown function sequence and firstly demonstrated that microsomal glutathione S-transferase 1 (MGST1), ATP synthase H^+^ transporting mitochondrial F_0_ complex subunit C1, selenoprotein S, mitochondrial IF1 protein, and mitochondrial succinyl-CoA synthetase alpha subunit were participated in mitochondria metabolism, functional and structural damage process caused by chronic fluorosis.

Membrane-bound microsomal GST (MGST1), genetically different from the cytosolic GSTs, is a homotrimer with one thiol group (Cys49) per subunit and is activated by thiol alkylation, by disulfide bond formation or sulfenic acid formation, and by other mechanisms [10, 12–15]. mtMGST1, a similar type of microsomal MGST1, is also activated by modification of thiol to a mixed disulfide bond (S-glutathionylation) and by a disulfide-linked mtMGST1 dimer in oxidative stress in vivo and in vitro. It was also clarified that the activation of mtMGST1 contributes to cytochrome c release from mitochondria [16]. MGST1 is activated by oxidative stress, and mtMGST1 is activated through mixed disulfide bond formation that contributes to cytochrome c release from mitochondria through the mitochondrial permeability transition (MPT) pore [16]. Fluoride can induce an increase in the release of cytochrome c from the mitochondria to the cytosol and straightly enter in mitochondria metabolism. In addition, NADPH, and ATP-depended activation of MGST1, which showed the relationship between MGST1 and cellular respiration [17]. According to these references, we could demonstrate that MGST1 participated in the cellular effects by fluorosis on cellular respiration and inner membrane permeability and membrane potential.

ATP synthase H^+^ transporting mitochondrial F_0_ complex subunit C1 is an isoform of mtATP synthase subunit 9. F_0_F_1_-ATP synthase, also known as ATP synthase, is composed of three parts: the catalytic sector F_1_, consisting of five subunits (3*α*; 3*β*; *γ*; *δ*; *ɛ*), the H^+^ translocating sector F_0_, and a stalk connecting F_1_ with F_0_, both consisting of a variable number of subunits [18–20]. In the F_0_F_1_-ATP synthase complex, in addition to the F_1_-*γ* and the F_0_I-PVP (b) subunits [19], also the oligomycin-sensitivity-conferring protein (OSCP) is involved in the coupling of the F_1_ catalytic activity to transmembrane proton translocation by F_0_ [21]. It has been shown that the F_0_F_1_-ATP synthase represents one of the sites of T3 action [22, 23]. It is no doubt that ATP synthase expression will be in differential level between fluorosis rat and control rat. Each process including cellular respiration, ROS generation, inner membrane permeability, calcium and phosphate transportation, glucose transport, and cell apoptosis need ATP synthase and energy. Obviously we should know its importance in resistant to fluorosis.

 Another gene we screened, mitochondrial IF1 protein, is associated to ATPase activity. IF1 is present in animals [24, 25], yeasts [26, 27], and plants [28, 29]. Its structure is essentially *α*-helical [30]. Pioneering investigations have shown that bovine IF1 (bIF1) lacking the first 16 residues after partial enzymatic digestion retains the capacity to inhibit ATPase activity [31]. It has been later reported that the minimal inhibitory sequence consists of residues 22–46 [32] or 14–47 [23] in bovine and of residues 17–41 in yeasts [32]. Deletion of the first 17 residues in mammal IF1 resulted in a still inhibitory peptide but with a very low affinity for ATP synthase, whereas conflicting results were reported concerning the removal of the first 21 residues [32–34]. Importantly, the visible part in N-terminus of IF1 (residues 8–21) appears wrapped around the *γ* subunit and also interacting with the *α*-subunit. This finding raises the problem of the role of the Nter part of IF1 in the different steps of the inhibitory process. IF1 peptides partially truncated in Nter have been shown to inhibit ATPase activity after several minutes of incubation [33, 34], but in vivo the inhibitory peptide very rapidly responds to pmf variations, as shown by the absence of ATP hydrolysis activity immediately after adding an uncoupler to mitochondria [35]. IF1 appears to limit mitochondrial ROS generation, limiting autophagy which is increased by IF1 knockdown [36].

We screened mitochondrial succinyl-CoA synthetase alpha subunit (mtSCS), and its function is also correlated to ATPase activity. SCS is a Krebs cycle enzyme that catalyzes substrate-level phosphorylation in the forward direction and replenishes succinyl-CoA for ketone body catabolism and porphyrin biosynthesis in the reverse direction [37–39]. In mammals two isoforms of SCS have been identified: one specific for ADP/ATP and another specific for GDP/GTP. In some species (i.e., *Escherichia coli*), SCS is not specific, using either the ATP or GTP [39]. SCS*α* has a well-documented histidine phosphorylation site but has also been shown to bind phosphate in its dephosphorylated form as a means of stabilizing the complex [39]. Furthermore, SCS can generate ATP in the absence of a proton motive force in the inner membrane, potentially playing a role in maintaining matrix ATP levels under energy-limited conditions, such as transient hypoxia. Inorganic phosphate (Pi), a putative cytosolic signaling molecule, plays a multifaceted role in the regulation of mitochondrial metabolism. Pi can alter the free concentration of Mg^2+^ and Ca^2+^ ions, increase mitochondrial volume, influence the mitochondrial transition pore, and directly modify the activity of several Krebs cycle dehydrogenases [40–43].

Selenoprotein S has recently been described as an endoplasmic reticulum (ER) and plasma membrane-located selenoprotein involved in the physiologic adaptation to ER stress [44, 45]. The SELS gene is known to be expressed in a wide variety of tissues and cell types, including tissues important for glycemic control such as adipose tissue, muscle, and liver [44, 45]. Some studies have shown that SELS genotype is associated with circulating levels of proinflammatory cytokines, such as tumor necrosis factor-*α* (TNF-*α*) and interleukin-1*β* (IL-1*β*), suggesting that SELS plays a role in inflammation [46].

## 5. Conclusion

 Microsomal glutathione S-transferase 1 (MGST1), ATP synthase H^+^ transporting mitochondrial F_0_ complex subunit C1, selenoprotein S, mitochondrial IF1 protein, and mitochondrial succinyl-CoA synthetase alpha subunit were participated in mitochondria metabolism, functional and structural damage process caused by chronic fluorosis. This information will be very helpful for understanding the molecular mechanisms of fluorosis.

## Figures and Tables

**Table 1:** Differential expression genes of DDRT-PCR for fluorosis in rat liver.

No.	GenBank accession number	bp	Copies	BLAST results	Score	*E* value	Identities
(1)	NM_134349.3	183	1	Microsomal glutathione S-transferase 1	307 bits (166)	1*e*−80	170/172 (99%)
(2)	NM_012820.1	324	1	Acyl-CoA synthetase long-chain family member	540 bits (292)	2*e*−150	297/299 (99%)
(3)	New EST	176	1	No significant similarity found	/	/	/
(4)	AI029143.1	317	1	UI-R-C0-ip-g-08-0-UI	549 bits (297)	4*e*−153	304/307 (99%)
(5)	NM_017311.1	266	1	ATP synthase, H^+^ transporting, mitochondrial F_0_ complex, subunit C1	444 bits (240)	2*e*−121	240/240 (100%)
(6)	NM_012582.2	332	1	Haptoglobin	586 bits (317)	3*e*−164	319/320 (99%)
(7)	NM_173120.2	318	2	Selenoprotein S	558 bits (302)	6*e*−156	309/312 (99%)
(8)	L07806.1	483	1	Mitochondrial IF1 protein	761 bits (412)	0.0	412/412 (100%)
(9)	New EST	283	1	No significant similarity found	/	/	/
(10)	NM_012820.1	196	1	Acyl-CoA synthetase long-chain family member	540 bits (292)	2*e*−150	297/299 (99%)
(11)	New EST	311	1	No significant similarity found	/	/	/
(12)	J03621.1	455	1	Mitochondrial succinyl-CoA synthetase alpha subunit	797 bits (431)	0.0	433/434 (99%)

## References

[1] Barbier O, Arreola-Mendoza L, Del Razo LM (2010). Molecular mechanisms of fluoride toxicity. *Chemico-Biological Interactions*.

[2] Weinstein LH, Davidson A (2004). *Fluorides in the Environment. Effects on Plants and Animals*.

[3] Urbansky ET (2002). Fate of fluorosilicate drinking water additives. *Chemical Reviews*.

[4] Karube H, Nishitai G, Inageda K, Kurosu H, Matsuoka M (2009). NaF activates MAPKs and induces apoptosis in odontoblast-like cells. *Journal of Dental Research*.

[5] Zhang Y, Li W, Chi HS, Chen J, DenBesten PK (2007). JNK/c-Jun signaling pathway mediates the fluoride-induced down-regulation of MMP-20 in vitro. *Matrix Biology*.

[6] Zhang M, Wang A, Xia T, He P (2008). Effects of fluoride on DNA damage, S-phase cell-cycle arrest and the expression of NF-*κ*B in primary cultured rat hippocampal neurons. *Toxicology Letters*.

[7] Anuradha CD, Kanno S, Hirano S (2001). Oxidative damage to mitochondria is a preliminary step to caspase-3 activation in fluoride-induced apoptosis in HL-60 cells. *Free Radical Biology and Medicine*.

[8] Cittanova ML, Lelongt B, Verpont MC (1996). Fluoride ion toxicity in human kidney collecting duct cells. *Anesthesiology*.

[9] Dabrowska E, Balunowska M, Letko R, Szynaka B (2004). Ultrastructural study of the mitochondria in the submandibular gland, the pancreas and the liver of young rats, exposed to NaF in drinking water. *Roczniki Akademii Medycznej w Bialymstoku*.

[10] Imaizumi N, Miyagi S, Aniya Y (2006). Reactive nitrogen species derived activation of rat liver microsomal glutathione S-transferase. *Life Sciences*.

[11] Zhao S, Ooi SL, Pardee AB (1995). New primer strategy improves precision of differential display. *BioTechniques*.

[12] Hossain QS, Ulziikhishig E, Lee KK, Yamamoto H, Aniya Y (2009). Contribution of liver mitochondrial membrane-bound glutathione transferase to mitochondrial permeability transition pores. *Toxicology and Applied Pharmacology*.

[13] Andersson C, Mosialou E, Weinander R, Morgenstern R (1994). Enzymology of microsomal glutathione S-transferase. *Advances in Pharmacology*.

[14] Aniya Y, Anders MW (1989). Regulation of rat liver microsomal glutathione S-tranferase activity by thiol/disulfide exchange. *Archives of Biochemistry and Biophysics*.

[15] Shimoji M, Aniya Y, Morgenstern R, Awasthi YC (2007). Activation of microsomal glutathione transferase 1. *Toxicology of Glutathione Transferases*.

[16] Lee KK, Shimoji M, Hossain QS, Sunakawa H, Aniya Y (2008). Novel function of glutathione transferase in rat liver mitochondrial membrane: role for cytochrome c release from mitochondria. *Toxicology and Applied Pharmacology*.

[17] Rinaldi R, Aniya Y, Svensson R (2004). NADPH dependent activation of microsomal glutathione transferase. *Chemico-Biological Interactions*.

[18] Mangiullo R, Gnoni A, Damiano F (2010). 3,5-diiodo-L-thyronine upregulates rat-liver mitochondrial F_0_F_1_-ATP synthase by GA-binding protein/nuclear respiratory factor-2. *Biochimica et Biophysica Acta*.

[19] Papa S, Guerrieri F, Zanotti F, Fiermonte M, Capozza G, Jirillo E (1990). The *γ* subunit of F_1_ and the PVP protein of F_0_ (F_0_I) are components of the gate of mitochondrial F_0_F_1_H+-ATP synthase. *FEBS Letters*.

[20] Berden JA, Hartog AF (2000). Analysis of the nucleotide binding sites of mitochondrial ATP synthase provides evidence for a two-site catalytic mechanism. *Biochimica et Biophysica Acta*.

[21] Xu T, Zanotti F, Gaballo A, Raho G, Papa S (2000). F_1_ and F_0_ connections in the bovine mitochondrial ATP synthase. The role of the of *α* subunit N-terminus, oligomycin-sensitivity conferring protein (OCSP) and subunit d. *European Journal of Biochemistry*.

[22] Hafner RP, Brown GC, Brand MD (1990). Thyroid-hormone control of state-3 respiration in isolated rat liver mitochondria. *Biochemical Journal*.

[23] Guerrieri F, Kalous M, Adorisio E (1998). Hypothyroidism leads to a decreased expression of mitochondrial F_0_F_1_- ATP synthase in rat liver. *Journal of Bioenergetics and Biomembranes*.

[24] Andrianaivomananjaona T, Moune-Dimala M, Herga S, David V, Haraux F (2011). How the N-terminal extremity of Saccharomyces cerevisiae IF1 interacts with ATP synthase: a kinetic approach. *Biochimica et Biophysica Acta*.

[25] Pullman ME, Monroy GC (1963). A naturally occurring inhibitor of mitochondrial adenosine. *The Journal of Biological Chemistry*.

[26] Satre M, de Jerphanion MB, Huet J, Vignais PV (1975). ATPase inhibitor from yeast mitochondria. Purification and properties. *Biochimica et Biophysica Acta*.

[27] Matsubara H, Hase T, Hashimoto T, Tagawa K (1981). Amino acid sequence of an intrinsic inhibitor of mitochondrial ATPase from yeast. *Journal of Biochemistry*.

[28] Norling B, Tourikas C, Hamasur B, Glaser E (1990). Evidence for an endogenous ATPase inhibitor protein in plant mitochondria. Purification and characterization. *European Journal of Biochemistry*.

[29] Polgreen KE, Featherstone J, Willis AC, Harris DA (1995). Primary structure and properties of the inhibitory protein of the mitochondrial ATPase (H+-ATP synthase) from potato. *Biochimica et Biophysica Acta*.

[30] Cabezón E, Runswick MJ, Leslie AGW, Walker JE (2002). The structure of bovine IF1, the regulatory subunit of mitochondrial F-ATPase. *EMBO Journal*.

[31] Dianoux AC, Tsugita A, Klein G, Vignais PV (1982). Effects of proteolytic fragmentations on the activity of the mitochondrial natural ATPase inhibitor. *FEBS Letters*.

[32] Stout JS, Partridge BE, Dibbern DA, Schuster SM (1993). Peptide analogs of the beef heart mitochondrial F1-ATPase inhibitor protein. *Biochemistry*.

[33] van Raaij MJ, Orriss GL, Montgomery MG (1996). The ATPase inhibitor protein from bovine heart mitochondria: the minimal inhibitory sequence. *Biochemistry*.

[34] Lebowitz MS, Pedersen PL (1996). Protein inhibitor of mitochondrial ATP synthase: relationship of inhibitor structure to pH-dependent regulation. *Archives of Biochemistry and Biophysics*.

[35] Venard R, Brèthes D, Giraud MF, Vaillier J, Velours J, Haraux F (2003). Investigation of the role and mechanism of IF1 and STF1 proteins, twin inhibitory peptides which interact with the yeast mitochondrial ATP synthase. *Biochemistry*.

[36] Campanella M, Seraphim A, Abeti R, Casswell E, Echave P, Duchen MR (2009). IF1, the endogenous regulator of the F_1_F_o_-ATPsynthase, defines mitochondrial volume fraction in HeLa cells by regulating autophagy. *Biochimica et Biophysica Acta*.

[37] Phillips D, Aponte AM, French SA, Chess DJ, Balaban RS (2009). Succinyl-CoA synthetase is a phosphate target for the activation of mitochondrial metabolism. *Biochemistry*.

[38] Kaufman S, Gilvarg C, Cori O, Ochoa S (1953). Enzymatic oxidation of alpha-ketoglutarate and coupled phosphorylation. *Journal of Biological Chemistry*.

[39] Fraser ME, James MNG, Bridger WA, Wolodko WT (2000). Phosphorylated and dephosphorylated structures of pig heart, GTP-specific succinyl-CoA synthetase. *Journal of Molecular Biology*.

[40] Holt SJ, Riddle DL (2003). SAGE surveys C. elegans carbohydrate metabolism: evidence for an anaerobic shift in the long-lived dauer larva. *Mechanisms of Ageing and Development*.

[41] Weinberg JM, Venkatachalam MA, Roeser NF, Nissim I (2000). Mitochondrial dysfunction during hypoxia/reoxygenation and its correction by anaerobic metabolism of citric acid cycle intermediates. *Proceedings of the National Academy of Sciences of the United States of America*.

[42] Basso E, Petronilli V, Forte MA, Bernardi P (2008). Phosphate is essential for inhibition of the mitochondrial permeability transition pore by cyclosporin A and by cyclophilin D ablation. *Journal of Biological Chemistry*.

[43] Zavala JSR, Pardo JP, Sanchez RM (2000). Modulation of 2-oxoglutarate dehydrogenase complex by inorganic phosphate, Mg2+, and other effectors. *Archives of Biochemistry and Biophysics*.

[44] Olsson M, Olsson B, Jacobson P (2011). Expression of the selenoprotein S (SELS) gene in subcutaneous adipose tissue and SELS genotype are associated with metabolic risk factors. *Metabolism: Clinical and Experimental*.

[45] Kim KH, Gao Y, Walder K, Collier GR, Skelton J, Kissebah AH (2007). SEPS1 protects RAW264.7 cells from pharmacological ER stress agent-induced apoptosis. *Biochemical and Biophysical Research Communications*.

[46] Curran JE, Jowett JBM, Elliott KS (2005). Genetic variation in selenoprotein S influences inflammatory response. *Nature Genetics*.

